# Whole-Genome Analysis of a Novel Multidrug-Resistant *Escherichia coli* Strain from Dairy Calves in Northeast China: Mechanisms of Antibiotic Resistance and Biofilm Formation

**DOI:** 10.3390/biology14091257

**Published:** 2025-09-12

**Authors:** Xuanpan Ding, Qiuyue An, Huijie Kang, Siyao Li, Shuai Zhang, Haotian Yang, Xinyi Dou, Yaxin Ji, Yuan Zhao, Honggang Fan

**Affiliations:** Heilongjiang Key Laboratory for Laboratory Animals and Comparative Medicine, College of Veterinary Medicine, Northeast Agricultural University, Harbin 150030, China; a1910225288@163.com (X.D.); m15383052857@163.com (Q.A.); hj_kang8436@163.com (H.K.); dylisiyao@163.com (S.L.); zhangshuai19900322@gmail.com (S.Z.); yanghaotian95@163.com (H.Y.); douxinyi0805@163.com (X.D.); jingjing20001129@163.com (Y.J.); 16645266261@163.com (Y.Z.)

**Keywords:** multidrug-resistant *Escherichia coli*, genomics analysis, biofilm formation, zoonotic transmission, agricultural antimicrobial use

## Abstract

The use of antibiotics is so widespread in animal husbandry, but negligent management and lack of policies often lead to the massive use of antibiotics on farms. In this study, we collected cases of epidemic calf diarrhea in northeastern China and isolated a new strain of multidrug-resistant *Escherichia coli* (MDR-*E. coli*). In order to explore the information of this pathogen in detail, we used whole-genome sequencing to determine the genome sequence, and explored in detail the resistance, pathogenicity, genetic evolution and other biological processes of the strain through bioinformatics analysis. The results showed that the *E. coli* isolated in this study was a new multidrug-resistant strain with a large number of drug resistance genes (77) and virulence genes (84), including a circular chromosome and five circular plasmids, which are basically impossible to treat by currently commonly used antibiotics. The findings of this study suggest that the prolonged misuse of antibiotics in agricultural settings may contribute to the development of antibiotic-resistant strains of *E. coli*. This, in turn, has the potential to trigger outbreaks of antibiotic-resistant bacterial diarrhea, leading to substantial economic losses and posing significant public health risks. These results underscore the necessity for the judicious use of antibiotics and will inform the development of pertinent policies and regulations.

## Simple Summary

Excessive antibiotic use in agriculture is fostering hard-to-treat pathogenic bacteria, endangering animal and human health. This study investigated a severe calf diarrhea outbreak caused by a new *Escherichia coli* strain resistant to most antibiotics. Genetic analysis revealed numerous drug resistance and pathogenicity genes, enabling antibiotic expulsion and biofilm formation. Mouse models confirmed its severe disease potential. Our research highlights the dangers of agricultural antibiotic misuse, leading to nearly untreatable superbugs, and stresses the urgent need for more prudent antibiotic use to protect public health.

## 1. Introduction

The emergence and global spread of multidrug-resistant MDR-*E. coli* strains represent a significant threat to both veterinary and human medicine [1,2]. While commensal *E. coli* is a component of the normal gut microbiota, pathogenic variants have developed sophisticated resistance mechanisms and virulence factors that enable them to cause severe infections [3]. Of particular concern is the agricultural sector, where the excessive use of antibiotics has become a major driver of antimicrobial resistance (AMR) evolution [4]. Recent studies project that antimicrobial consumption in food animals will increase to 105,596 tons globally by 2030, potentially accelerating the emergence of untreatable bacterial strains [5]. Calves are particularly susceptible to *E. coli* infections, which result in severe diarrhea and high mortality, leading to significant economic losses in dairy farms. Monitoring resistant strains in calves is crucial for early intervention and the prevention of zoonotic transmission to humans and other animals.

Whole genome sequencing (WGS) has significantly enhanced our capacity to characterize bacterial pathogens with unprecedented precision [6]. This technology facilitates a comprehensive analysis of resistance gene repertoires, virulence determinants, and mobile genetic elements that promote horizontal gene transfer [7]. Several databases, such as the Comprehensive Antibiotic Resistance Database (CARD) and the Virulence Factor Database (VFDB), have been established to annotate these genomic features [8,9,10]. Despite these advancements, a critical knowledge gap persists concerning the genomic architecture of farm-associated MDR-*E. coli* and its correlation with clinical treatment failures.

In this study, we utilized a combination of second- and third-generation sequencing technologies to characterize a novel MDR-*E. coli* strain isolated from a high-mortality calf diarrhea outbreak. Through comparative genomics, functional annotation, and phenotypic validation, we: (i) identified a unique combination of 77 resistance genes and 84 virulence factors; (ii) elucidated the predominant resistance mechanisms, including efflux pumps and biofilm formation; and (iii) demonstrated the strain’s pathogenic potential through murine infection models. Our findings provide critical insights into how agricultural antibiotic use drives the evolution of pan-resistant pathogens with zoonotic potential.

## 2. Materials and Methods

### 2.1. Sample Collection and Test Materials

Between September 2023 and June 2024, fecal samples were obtained from 67 calves exhibiting diarrhea in Bei’an City, located in northeastern China (126° E, 47° N), for the purposes of bacterial isolation and identification. The incidence of diarrhea among the calves at the farm was approximately 24.3%, with clinical symptoms including yellowish-green watery diarrhea with a foul odor, sunken eye sockets, dehydration, and a mortality rate of approximately 8.7%. Prior to sample collection, the calves were restrained, and the area surrounding the anus was disinfected using 0.1% benzalkonium bromide. Fecal samples were then collected directly from the rectum using sterile cotton swabs. Animal experiments were conducted using eight-week-old female SPF Kunming mice, weighing between 18 and 20 g, sourced from Northeast Agricultural University (Harbin, China). Bacterial isolation, amplification, and antibiotic sensitivity testing were performed using nutrient broth medium, Müller–Hinton broth, and MacConkey agar, among other materials provided by Servicebio (Wuhan, China). Various antibiotic tests were conducted, with detailed information available at https://www.solarbio.com/ (accessed on 15 February 2025) and in the Supplementary Materials, specifically in Table S1, which outlines the separation and identification results. Incubators (Aolai, Jinan, China), bacterial genomic extraction kits (JINGMEI BIOTECHNOLOGY, Nanjing, China), PCR amplifiers (Pultton Technology, Torrington, CT, USA), gel imaging systems (APExBIO, Houston, TX, USA), field emission scanning electron microscopes (Phenom, Rotterdam, South Holland, Netherlands), etc., were used for genomic detection and phenotypic verification.

### 2.2. Bacterial Isolation, Identification and Antibiotic Susceptibility Testing

To isolate pathogenic bacteria, selective culture media were utilized, and individual colonies were subsequently chosen for further streaking and purification. After three rounds of purification, bacterial cultures were established using liquid culture media. The cultured bacterial suspension underwent centrifugation to collect the bacterial pellet. Bacterial DNA was extracted using a bacterial genomic DNA extraction kit, and polymerase chain reaction (PCR) amplification was performed with universal bacterial 16S rRNA primers. The amplification products were analyzed via electrophoresis on a 1% agarose gel, and the band positions were visualized using a gel imaging system. Upon confirming the suitability of the amplification products, 16S rRNA sequencing was conducted. The sequencing results were subsequently analyzed utilizing the GenBank module of the National Center for Biotechnology Information (NCBI), adhering to the guidelines established by the Clinical and Laboratory Standards Institute (CLSI) [11]. The antibiotic susceptibility of pathogenic bacteria was evaluated through the double dilution method. In this protocol, a bacterial culture with a concentration of 1 × 10^6^ CFU/mL was introduced into the wells of a 96-well plate, following a two-fold serial dilution of the antibiotic. The ninth well, which contained no bacterial solution, served as the negative control, while the tenth well, lacking antibiotic, functioned as the positive control. Observations were conducted after a 24 h incubation period at 37 °C. Wells that exhibited no significant bacterial growth were designated as the minimum inhibitory concentration (MIC).

### 2.3. Whole Genome Sequencing of Representative Strain

Following antibiotic sensitivity testing, a novel MDR-*E. coli* strain (BA1) was isolated for comprehensive genome-wide sequencing analysis. The BA1 strain was cultured to the logarithmic growth phase in Luria–Bertani liquid medium and subsequently subjected to centrifugation at 4 °C for 10 min at 4000× *g* to collect the bacterial pellet, after which the supernatant was discarded. WGS was then conducted. Library construction and quality assessment were carried out using the PacBio and Illumina platforms. Sequencing was performed on the PacBio Sequel and Illumina NovaSeq PE150 systems once the library quality was confirmed. The genome assembly was executed using SOAPdenovo2 software (Version 2.04, https://github.com/aquaskyline/SOAPdenovo2, accessed on 21 February 2025), and the assembly results were refined using GapCloser software (Version 1.2.1, https://sourceforge.net/, accessed on 21 February 2025) to achieve a high-quality complete bacterial genome sequence. Core genome multilocus sequence typing (cgMLST) was analyzed via the EnteroBase online platform (EnteroBase 2024, accessed on 21 February 2025), and the phylogenetic tree was constructed using Grapetree (Version 3.0.4, accessed on 21 February 2025).

### 2.4. Gene Prediction and Functional Annotation

Coding sequences (CDS) within the genome were identified utilizing the Glimmer (http://ccb.jhu.edu/software/glimmer/index.shtml, accessed on 23 February 2025), GeneMarkS (http://topaz.gatech.edu/GeneMark, accessed on 23 February 2025), and Prodigal (https://github.com/hyattpd/Prodigal, accessed on 23 February 2025) software tools. Chromosomal genomes were specifically predicted using Prodigal, while plasmid genomes were delineated using GeneMarkS. The prediction of tRNAs present in the genome was conducted with the tRNAscan-SE v2.0 software (http://trna.ucsc.edu/software/, accessed on 23 February 2025). Furthermore, the identification of rRNAs within the genome was executed using Barrnap software (Version 0.9, https://github.com/tseemann/barrnap, accessed on 23 February 2025), which facilitated the acquisition of species, location, and sequence information for all rRNAs in the genome of each sample. The predicted coding genes underwent functional annotation through collaboration with six major databases: the Non-Redundant Protein Database (NR), the Swiss-Prot database, the Pfam database, EggNOG (Evolutionary Genealogy of Genes: Non-supervised Orthologous Groups), the Gene Ontology (GO) annotation, and the Kyoto Encyclopedia of Genes and Genomes (KEGG) database. Functional annotation was primarily based on protein sequence comparisons. Each gene sequence was systematically compared against these databases to acquire the relevant functional annotation information. Additionally, the genomic circular map for a single sample was generated using the CGview software (Gene annotation and map generation time: 23 February 2025).

### 2.5. Bioinformatics Analysis

#### 2.5.1. Analysis of Virulence Genes and Resistance Genes

After comparing BA1 genome with CARD and VFDB, respectively, drug resistance genes and virulence genes were obtained [12,13]. The comparison results were screened to obtain genes with high confidence (screening conditions: confidence > 80%, coverage > 80%). Protein interactions networks of resistance genes and virulence factors in BA1 were mapped based on the STRING (https://cn.string-db.org/, accessed on 27 February 2025) database and Cytoscape tool (Version 3.10.1, accessed on 27 February 2025), with *E. coli* K12 MG1655 as the reference and the minimum protein interactions score set to a high confidence level of 0.700.

#### 2.5.2. Pathogenic Bacteria Secretion System and Protein Analysis

The analysis of secretory systems provides information on the types and quantities of secretory systems in samples, the genes involved, their functions, and related metabolic pathways. Secretory proteins are synthesized within the cell and secreted externally. SignalP (http://www.cbs.dtu.dk/services/SignalP, accessed on 23 February 2025) [14] predicts secreted proteins by identifying protein-coding genes with signal peptides lacking transmembrane structures. Secretion systems (T1SS-T6SS) and their genetic components are identified using MacSyFinder (v2.0, accessed on 23 February 2025) with specific models. SignalP (v6.0) analyzes coding sequences for Gram-negative bacterial signal peptides, while EffectiveDB screens effector proteins for systems like T3SS/T6SS. Functional annotation of secreted proteins and secretion system components was performed by BLASTp (https://blast.ncbi.nlm.nih.gov/Blast.cgi, accessed on 23 February 2025) alignment against the VFDB and the NCBI NR, applying an *E*-value cutoff of 1 × 10^−5^ and minimum 70% coverage threshold.

#### 2.5.3. Transporter Protein Annotations

The Transporter Classification Database (TCDB, http://www.tcdb.org/, accessed on 23 February 2025) contains more than 10,000 non-redundant transport systems classified into 1322 families of transport proteins [15,16]. Transporter proteins were identified and classified using the TCDB. Predicted protein sequences were analyzed with BLASTp against TCDB (E-value < 1 × 10^−10^, coverage > 70%). Proteins were categorized into TCDB classes—such as Primary Active Transporters and Channels—based on TC numbers. Significant matches were confirmed using HMMER scans against TCDB’s HMMs. Only high-confidence predictions (confidence > 90%, coverage > 90%) were used for further analysis.

#### 2.5.4. Analysis of Pathogen–Host Interactions

The Pathogen Host Interactions [17] (PHI) database contains experimentally validated content derived from fungal, oomycete, and bacterial pathogens, infecting hosts including animals, plants, fungi, and insects. The pathogen-host interaction gene annotation profiles were obtained and statistically compared to the pathogenic bacteria database, and information on pathogen-host interaction-related genes contained in each genome was obtained through PHI database annotation. The comparison results were screened to obtain genes with high confidence (screening conditions: confidence > 70%, coverage > 80%).

### 2.6. Metabolic System Analysis

Metabolic system analysis included carbohydrate-active enzyme annotation and secondary metabolite synthesis gene cluster analysis. Carbohydrate-active enzymes were categorized into six protein families, including glycoside hydrolases (GHs), glycosyltransferases (GTs), polysaccharide lyases (PLs), carbohydrate esterases (CEs), carbohydrate-binding modules (CBMs), and auxiliary oxidoreductases (AAs), based on similarity of amino acid sequences in the structural domains of the proteins. Annotation analysis was performed using the Carbohydrate Active Enzyme Database [18]. (CAZy, http://www.cazy.org/, accessed on 23 February 2025). The comparison results were screened to obtain genes with high confidence (screening conditions: coverage > 80%).

### 2.7. Mobile Genetic Elements and Transferability Analysis

To assess the potential for zoonotic transmission of resistance genes, the genome of BA1 was comprehensively analyzed for Mobile Genetic Elements (MGEs). Insertion Sequences (IS), transposons (Tn), and integrons were identified using MobileElementFinder (v1.0.3, accessed on 21 August 2025) [19] and the ISfinder database. Plasmid replicon types and plasmid MLST were determined using PlasmidFinder (v2.1, accessed on 21 August 2025) and pMLST (v2.0, accessed on 21 August 2025) to assess similarity to known conjugative plasmids [20]. Furthermore, plasmids harboring resistance genes were compared via BLASTn (accessed on 21 August 2025) against the NCBI nucleotide database to evaluate their homology with plasmids from human clinical strains.

### 2.8. qRT-PCR Validation

Based on the analysis of drug resistance and virulence genes using the STRING database, genes corresponding to significant nodes were selected for quantitative reverse transcription PCR (qRT-PCR) amplification. The *E. coli* standard strain ATCC25922 served as the control group. The PCR mixture comprised 20 μL of 2 × Taq Master Mix, 16 μL of double-distilled water (ddH_2_O), 1 μL each of the forward and reverse primers, and 2 μL of template DNA. The thermal cycling conditions were as follows: initial denaturation at 95 °C for 3 min, followed by 34 cycles of denaturation at 95 °C for 30 s, annealing at 58 °C for 30 s, and extension at 72 °C for 1 min, with a final extension step at 72 °C for 5 min [21].The bacterial 16S primer was used as the internal reference gene.

### 2.9. Biofilm Formation Assay

This study undertook a comprehensive analysis of biofilm formation by the strain using staining techniques and scanning electron microscopy. A 10 μL aliquot of bacteria cultured to the logarithmic phase was added to 190 μL of nutrient broth medium and incubated at 37 °C for 24 h. Subsequently, the bacterial solution was aspirated from the wells and washed three times with PBS (pH 7.2–7.4). After drying, a 1% crystal violet solution was applied, followed by incubation at 37 °C for 20 min. The crystal violet solution was then aspirated, and the wells were washed three times with PBS. After drying, 250 μL of 95% ethanol was added, and the wells were incubated at 37 °C for 20 min. The absorbance was measured at 595 nm using a microplate reader [22]. A negative control group without bacterial solution and a control group using the standard strain ATCC25922 were also established. The adhesion and biofilm formation capabilities of the strains were assessed based on the obtained OD values. After culturing the strain on round cover slips for 24 h, fix it with a 2.5% glutaraldehyde solution at 4 °C for 4 h. The samples were then rinsed three times with 0.1 M phosphate buffer (pH 7.4) and dehydrated using a gradient of 30%, 50%, 70%, 80%, 90%, and 100% ethanol, each for 10 min. The samples were dried using a critical point dryer and gold-coated in an ion sputtering chamber. Finally, a field emission scanning electron microscope was used to observe and collect three-dimensional structural images of the biofilm under conditions of 5.0 kV acceleration voltage and 8–10 mm working distance [23,24].

### 2.10. Pathogenicity Test

Twelve mice were randomly assigned to either the control group or the experimental model group, with six mice in each group. The model group received an intraperitoneal injection of 0.1 mL of pathogenic bacteria at a concentration of 1 × 10^8^, while the control group was administered an equivalent volume of saline. After 24 h, the physiological conditions of the mice were observed and documented. Subsequently, the mice were euthanized for dissection to examine gross lesions and collect samples for regression experiments. Concurrently, the organs were harvested, fixed in 4% formaldehyde solution for 24 h, embedded in paraffin wax, and subjected to microscopic examination following hematoxylin and eosin (H&E) staining. In cases where mice succumbed within the 24 h period, dissection and sampling were conducted immediately post-mortem. Histological evaluation of each test was performed by two independent histologists who were blinded to the group assignments.

### 2.11. Data Analysis

Statistical analyses were performed using SPSS version 22.0 (Inc, Chicago, IL, USA). Data were analyzed by *t*-test (between two groups). Statistical graphs were generated using Prism version 5.0 (GraphPad software Inc, La Jolla, CA, USA). *p* < 0.05 indicated statistically significant differences.

## 3. Results

### 3.1. Morphology and Identification of BA1

The BA1 appeared as pink colonies on MacConkey’s medium, and after Gram staining microscopic examination, they appeared as red short bacilli with a length of about 1 μm. Based on 16S rRNA gene sequencing results, the isolate was identified as *E. coli*. The isolated strain showed resistance to all commonly used antibiotics tested. (The results show that Supplementary Materials—Separation and identification of results, Table S1).

### 3.2. Whole Genome Sequencing and cgMLST Typing

**Figure 1:**
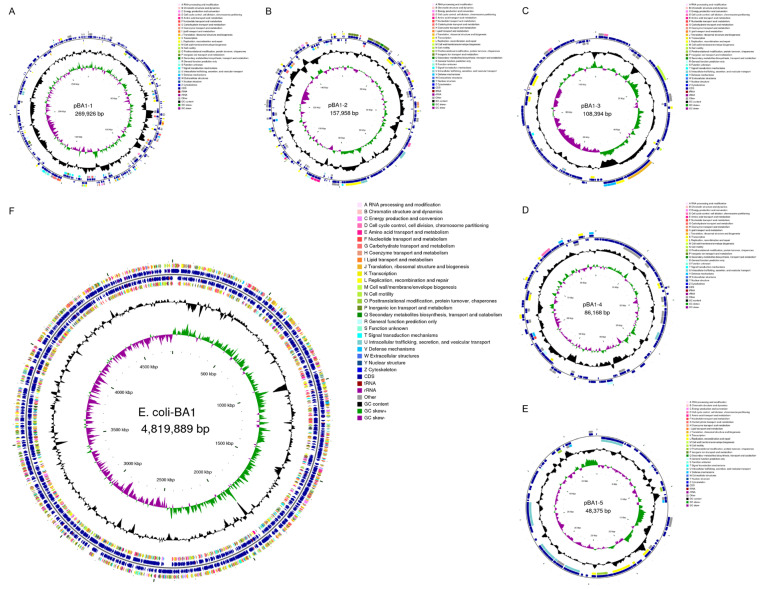
CG View Genome Circle Map. (**A**–**E**): plasmids 1–5; (**F**): chromosomes. The first and fourth circles of the circle diagram from the outside to the inside are the CDS on the positive and negative strands, and different colors indicate different COG functional classifications; the second and third circles are the CDS, tRNA, and rRNA on the positive and negative strands, respectively; the fifth circle is the GC content, and the outward portion indicates that the GC content of the region is higher than the average GC content of the whole genome, and the higher the peak indicates the higher the difference with the average GC content; the inward portion indicates that the GC content of the region is lower than the average GC content of the whole genome; and the sixth circle is the GC content, and the peak indicates the difference with the average GC content.

The quality assessment of whole genome sequencing revealed that the BA1 genome coverage exceeded 99%, with a contamination rate below 1%, indicating that the sequencing results were sufficiently robust for subsequent analyses. The BA1 genome comprised 6374 genes with a total length of 5,490,710 base pairs (bp), an average gene length of 739.19 bp, and a GC content of 51.54% (refer to Supplementary Materials: Separation and identification of results. Figure S3). Following sequence splicing and assembly, the BA1 strain genome was determined to consist of a circular chromosome measuring 4,819,889 bp with a GC content of 50.69%, alongside five circular plasmids (Figure 1). Comparative analysis using the PubMLST database classified the cgMLST typing of strain BA1 as cgST 23421.

### 3.3. Gene Prediction and Functional Annotation Results

**Figure 2:**
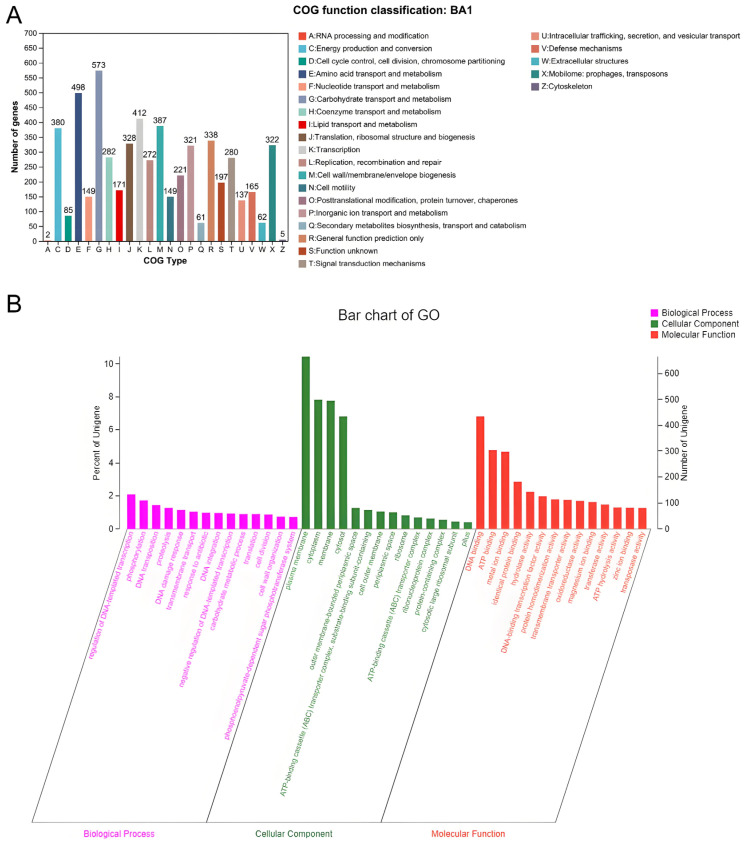
CGO and GO analysis results. (**A**): CGO annotation; horizontal coordinates represent different COG types and vertical coordinates represent the number of genes. Please see the legend on the right for the functional description of each COG type. (**B**): GO analysis; the horizontal coordinates represent the three major branches of GO—BP (Biological Process), CC (Cellular Component), MF (Molecular Function), and the further leve2 classification; the vertical coordinate represents the relative proportion of genes accounted for.

The analysis of predicted genomic non-coding RNAs (ncRNAs) identified 91 transfer RNAs (tRNAs), 22 ribosomal RNAs (rRNAs), including 5S, 16S, and 23S, and 285 small RNAs (sRNAs). Additionally, sixty tandem repeats were detected. Based on the comparison with the COG database, 5150 genes were assigned COG functional classifications. These include 1813 genes related to cellular processes and signaling, 1014 genes involved in information storage and processing, 2435 genes associated with metabolism, and 535 genes with functions that remain unknown. The predominant categories within the COG functional classification were genes involved in carbohydrate transport and metabolism, amino acid transport and metabolism, transcription, cell wall/cell membrane/transmembrane biosynthesis, and energy production and conversion (Figure 2A).

**Figure 3:**
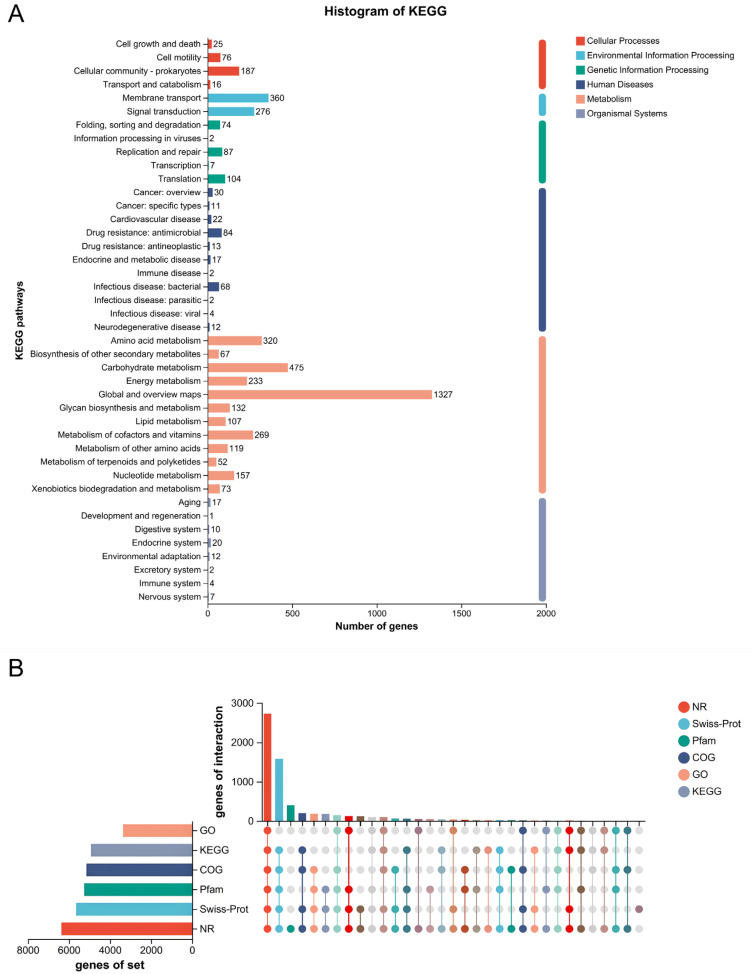
KEGG and pooled analysis results. (**A**): KEGG analysis; the vertical coordinate represents the level2 hierarchical classification of KEGG pathway, and the horizontal coordinate represents the number of genes under the annotation of this classification. Different column colors represent the level1 hierarchical classification of KEGG pathway. The rightmost bar indicates the number of genes under different level1 classifications. (**B**): Annotation summary; the horizontal bar on the left indicates the total number of genes annotated in each database; in the middle matrix, a single point indicates a gene specific to a database annotated in the left column; a line with multiple points indicates a gene that corresponds to a gene that is annotated in multiple databases; and the vertical bar indicates the number of genes specific to or shared by the database, respectively. The vertical bars indicate the corresponding number of unique/shared genes.

A total of 3369 genes were annotated with the results of GO analysis, including 2302 genes related to biological processes, 1979 genes related to cellular components, and 2744 genes related to molecular functions. Dominant terms included transmembrane transport and phosphorylation (BP); membrane, cytoplasm, and ABC transporter complex (CC); and ATP binding, metal ion binding, and DNA-binding transcription factor activity (MF). These results indicate robust capabilities in cellular transport, regulation, and energy-dependent functions, consistent with the strain’s adaptive and pathogenic profile. (Figure 2B). KEGG analysis showed that a total of 4932 genes were enriched in 263 metabolic pathways, among which the most involved pathways were metabolic pathway (ko01100) (1288 genes), biosynthesis of secondary metabolic products (ko01110) (497 genes), and microbial metabolism in different environments (ko01120) (383 genes) (Figure 3A). The number of genes annotated to the six major databases were (NR: 6368, Swiss-Prot: 5649, Pfam: 5259, COG: 5150, GO: 3369, KEGG: 4932) (Figure 3B).

### 3.4. Pathogenicity System Analysis

#### 3.4.1. Resistance Gene and Virulence Factor Analysis

**Figure 4:**
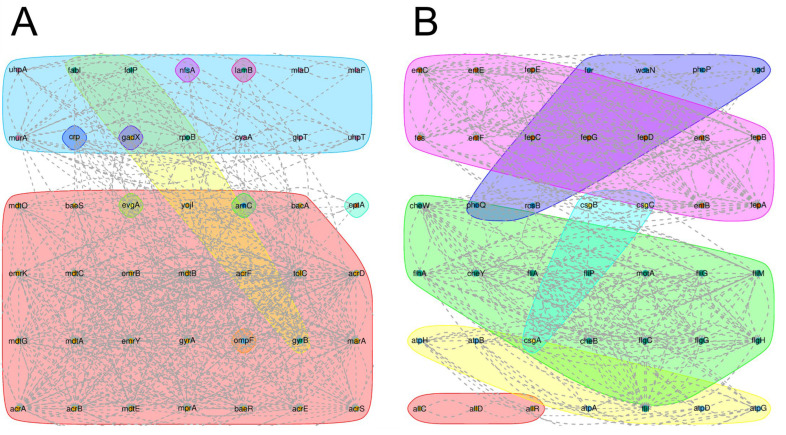
Resistance gene and Toxicity gene prediction results. (**A**): Results of STRING software analysis of drug resistance genes. (**B**): Results of STRING software analysis of toxicity genes. Colors represent sets with node values greater than 3.

Comparison with the CARD showed that BA1 carried 77 resistance genes. Statistically, the largest number of antibiotic resistance genes (ARG) are associated with antibiotic efflux, and the relevant efflux pumps include ABC-type efflux pumps, RND-type efflux pumps, MFS-type efflux pumps, etc. The most important ARGs are found to be associated with antibiotic efflux (Figure 4A,B). The virulence factors carried in the genome of BA1 strain were analyzed after comparing with the VFDB core database, and the results showed that there were 84 virulence factors on the chromosome and plasmid, which were categorized into 6 classes, namely, Nutritional/Metabolic factor, Effector delivery system, Adherence, Immune modulation, Motility, Regulation. These virulence factors are involved in various biological processes such as flagellar synthesis, bacterial motility, production of extracellular polysaccharides, intercellular aggregation, biofilm formation, cytotoxicity and invasion of cells.

#### 3.4.2. Secretory System and Protein Analysis

**Figure 5:**
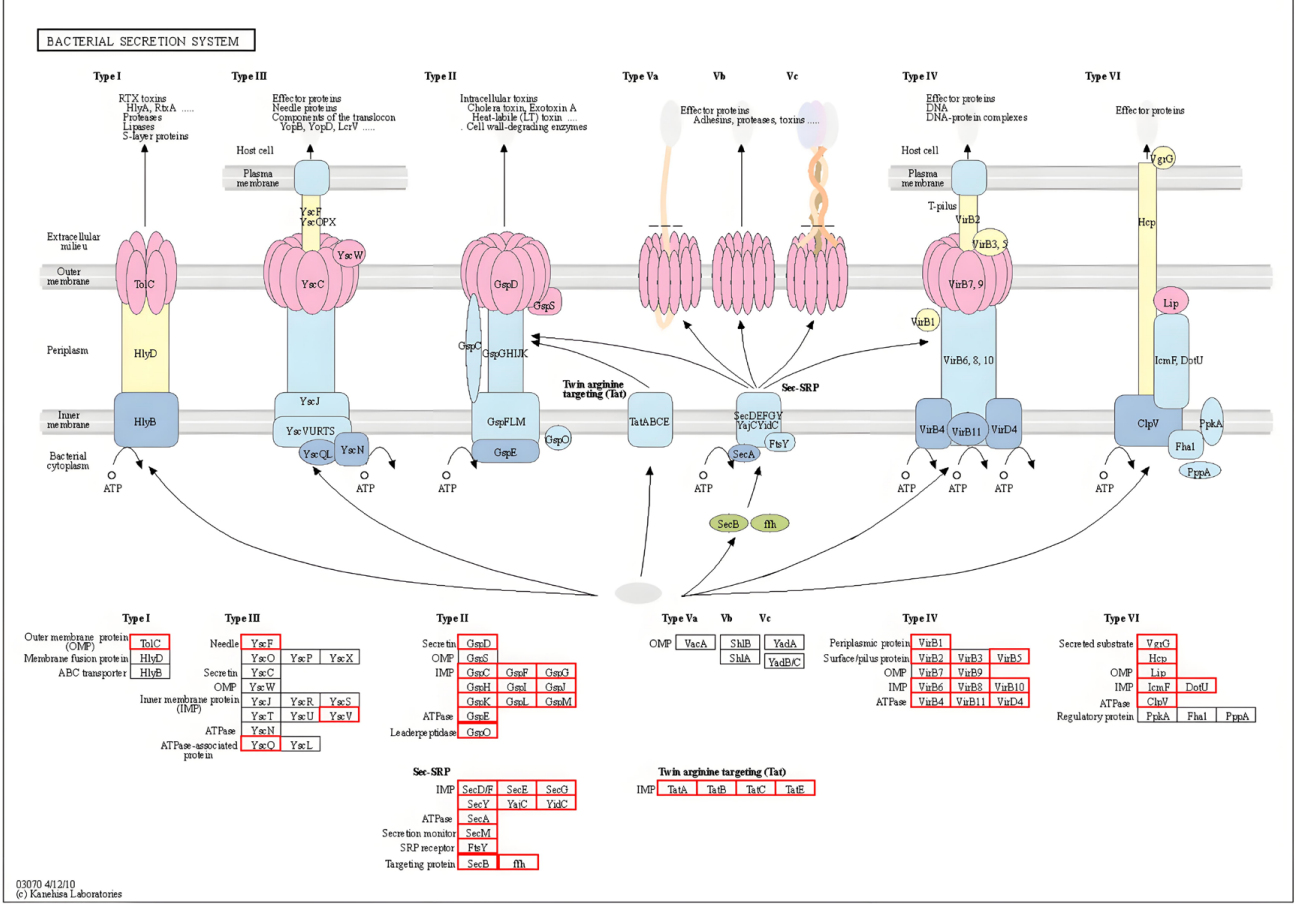
Secretory System Pathway Diagram. Red boxes: annotated to genes, protein visualization shown above.

A total of 77 genes were annotated to each of the six secretion systems, including 1 in Type I, 17 in Type II, 4 in Type III, 15 in Type IV, 17 in Type V, and 23 in Type VI. Meanwhile, 50 sensory proteins, and 7 heterotrimeric proteins were annotated. These proteins are mainly involved in biological processes such as antibiotic efflux, bacterial toxin production and secretion, bacterial adhesion and biofilm formation, bacterial invasion and colonization, DNA and protein secretion, and environmental stress response (Figure 5).

#### 3.4.3. Transporter Protein Analysis

**Figure 6:**
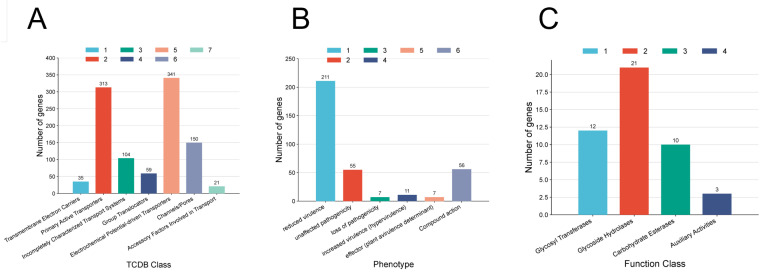
Pathogenic and metabolic systems analysis results. (**A**): Transporter protein analysis; (**B**): Results of host interaction analysis of pathogenic bacteria; (**C**): Metabolic system analysis results. The horizontal coordinate indicates the classification, and the vertical coordinate indicates the number of genes annotated to each classification.

A total of 1023 transporter protein-related genes were annotated by comparing the TCDB database. Among them, there are 313 Primary Active Transporters, 341 electrochemical potential-driven transporters and 150 Channels/Pores proteins. There are 104 proteins in completely characterized transport systems, 59 proteins in Group Translocators, 35 proteins in Transmembrane Electron Carriers and 21 proteins in accessory factors involved in transport (Figure 6A).

#### 3.4.4. Pathogen-Host Interaction Analysis

Annotation against the Pathogen Host Interactions (PHI) database identified a total of 347 genes in strain BA1 with high confidence that are implicated in interactions with host organisms. These genes were categorized based on their experimentally validated phenotypic effects upon mutation. The majority of these genes (211 genes) were associated with reduced virulence, indicating their crucial role in the bacterium’s ability to cause disease. A significant number of genes (55) were found to have unaffected pathogenicity when mutated, suggesting they are not essential for virulence under the tested conditions. Notably, 11 genes were linked to increased virulence—loss of these genes attenuates the pathogen, meaning their presence enhances BA1′s aggressiveness. Furthermore, 7 genes were classified as leading to a loss of virulence, and another 7 were annotated as plant avirulence determinants. Additionally, 16 genes were implicated in compound action, involved in responses to chemical or environmental stressors. (Figure 6B).

### 3.5. Metabolic System Analysis Results

After comparison with the Carbohydrate Active Enzyme Database, a total of 46 carbohydrate-active enzymes were annotated, of which 21 were Glycoside Hydrolases, 12 were Glycosyl Transferases, 10 were Carbohydrate Esterases, 3 were Auxiliary Activities. (Figure 6C).

### 3.6. Mobile Genetic Elements and Zoonotic Transmission Risk

Genomic analysis revealed a rich repertoire of MGEs in strain BA1, including 23 Insertion Sequences (IS) and 3 class 1 integrons. Critically, over 60% of the antibiotic resistance genes (ARGs) (47/77) were located on its five plasmids. Three of these plasmids (plasmid A, C, and E) carried key genes conferring resistance to β-lactams, quinolones, tetracyclines, and sulfonamides. Plasmid typing identified replicon types (e.g., *IncFIB, IncFII, IncX1*) commonly associated with conjugative plasmids in Enterobacteriaceae. Strikingly, BLASTn (Version 2.10.0) analysis revealed that plasmid A shared high sequence similarity (coverage > 90%, identity > 99%) with MDR plasmids found in *E. coli* strains isolated from human clinical infections, indicating that these multidrug-resistant plasmids are already being shared between animal and human reservoirs (Supplementary Materials: Separation and identification of results, Figures S1 and S2).

### 3.7. qRT-PCR Validation Results for Key Drug Resistance and Virulence Genes

**Figure 7:**
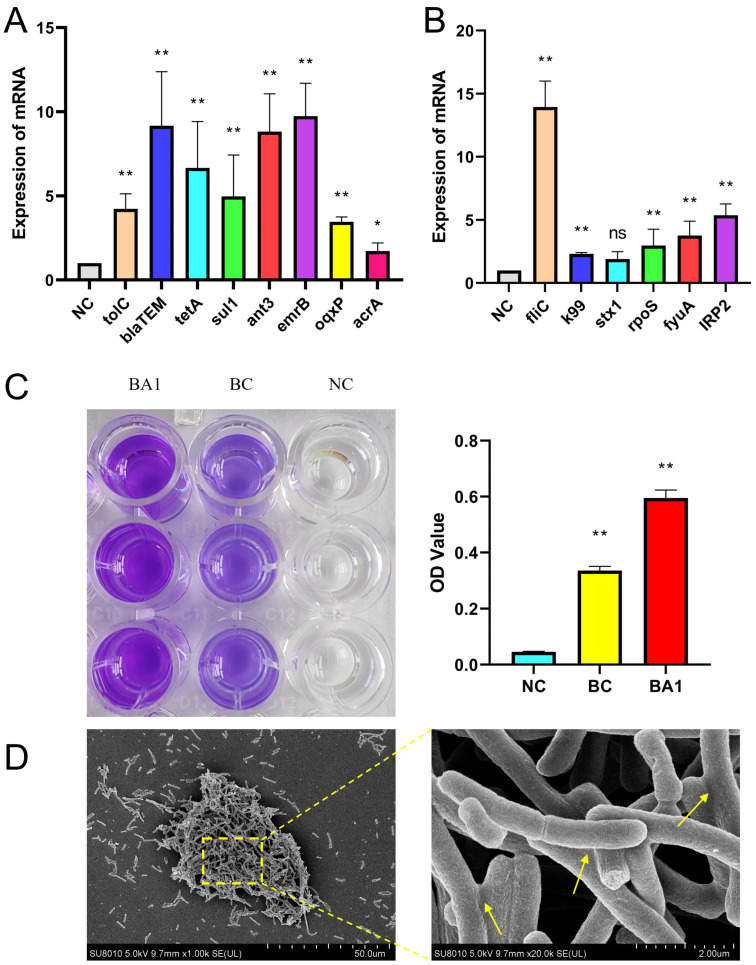
PCR validation and biofilm detection results. (**A**): mRNA expression of resistance genes; (**B**): mRNA expression of virulence genes; (**C**): results of biofilm assay. (**D**): The formation state of biofilm under scanning electron microscope, and the arrow indicates the bacteria that have been bonded. *: *p* < 0.05, **: *p* < 0.01, ns: *p* > 0.05.

To experimentally validate the genomic predictions of antibiotic resistance and virulence potential, the expression levels of key resistance and virulence genes were quantified using qRT-PCR and compared to the reference strain *E. coli* ATCC 25922. Significantly elevated expression was confirmed for a selection of critical genes. Among the antibiotic resistance genes, this included determinants conferring resistance to β-lactams (e.g., *blaTEM*), tetracyclines (e.g., *tetA*), sulfonamides (e.g., *sul2*) and antibiotic efflux (*tolC*), etc. Concurrently, the expression of pivotal virulence genes was also markedly upregulated. These included genes involved in adhesion, toxin production, and genes located on pathogenicity islands. The qRT-PCR results demonstrated that the mRNA expression levels of these targeted resistance and virulence genes in strain BA1 were significantly higher than those in the standard reference strain (*p* < 0.05). This transcriptional upregulation provides strong functional evidence that corroborates the genomic annotations, confirming that the extensive repertoire of resistance and virulence genes identified in silico is not only present but also actively and highly expressed in the BA1 isolate. This heightened expression phenotype is consistent with the strain’s observed multidrug-resistant and hypervirulent profile. (Figure 7A,B).

### 3.8. Biofilm Test Results

Bacterial biofilms significantly enhance antibiotic resistance by forming physical barriers and reducing metabolic activity, thereby hindering antibiotic penetration and inducing the production of persister cells. At the same time, horizontal gene transfer of ARGs regulated by quorum sensing within the biofilm (such as plasmid exchange) further accelerates the evolution of multidrug-resistant strains. After 24 h of incubation for each group, it was found that BA1 could clearly observe dark purple staining rings attached to the pore walls after staining after discarding the bacterial solution, while light purple rings were visible in the standard strain. The absorbance of BA1 after alcohol solubilization (OD = 595) was significantly higher than that of the standard strain (ATCC-25922) (*p* < 0.01) (Figure 7C). Scanning electron microscopy observations showed that the BA1 biofilm presented a complex three-dimensional structure, with tightly packed cells forming a multilayered structure, and the cells were interconnected through the extracellular matrix (Figure 7D).

### 3.9. Pathogenicity Test Results

**Figure 8:**
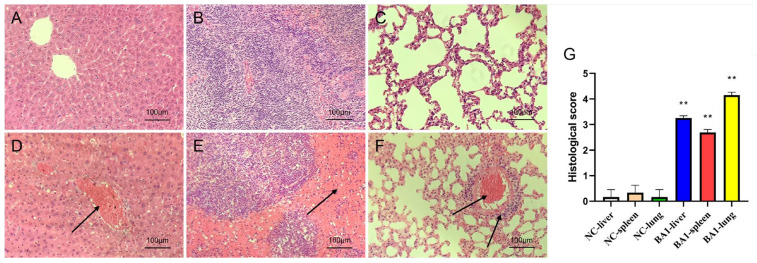
Animal model test results. (**A**): control liver, (**B**): control spleen, (**C**): control lung, (**D**): model liver, (**E**): model spleen, (**F**): model lung, (**G**): histologic score, **: *p* < 0.01. (H&E staining, ×100, Arrows: areas of pathologic changes).

After intraperitoneal injection of the pathogenic bacteria into mice, the mice were depressed, their appetites were cut off, they were lethargic, their coats were disorganized, and there was an increase in ocular secretion, and all of them died after an average time of 16 h. The results of regression experiments showed that BA1 was isolated from the blood and organs of the mice. Autopsy showed that the liver, lungs and spleen of the mice were congested and bruised and enlarged in different layers. Pathogenic BA1 was isolated from the blood of the mice, and tissue sections showed severe hemorrhage and bruising in the lungs, spleens, and livers of the model group, with a significant increase in histological scores (*p* < 0.01). The control group had no abnormalities in all indicators (Figure 8).

## 4. Discussion

The emergence of MDR-*E. coli* within agricultural ecosystems marks a critical juncture in the global AMR crisis, with profound implications extending beyond veterinary medicine into public health and food security [25,26,27]. Our study of the hypervirulent MDR-*E. coli* strain BA1, from a deadly calf diarrhea outbreak in northeastern China, shows a genome rich in resistance and virulence genes, challenging current views on microbial evolution and antibiotic use. BA1′s genome, with one chromosome and five plasmids, contains 77 ARGs and 84 virulence factors, demonstrating *E. coli*’s adaptability to antimicrobial pressure. Its resistance profile shows near-complete resistance to tested antibiotics, including critical ones like third-generation cephalosporins and fluoroquinolones. This consistency between genotype and phenotype not only affirms the value of WGS in resistance surveillance but also underscores the urgent need to reassess empirical treatment strategies in livestock production, where such strains are becoming increasingly common.

At the core of BA1′s resistance mechanisms is a complex network of efflux pumps, primarily comprising RND-type (e.g., *acrAB-tolC*), ABC-type, and MFS-type systems, which together constitute over 50% of its antibiotic resistance genes (ARGs). These systems, frequently overexpressed in response to antibiotic exposure, facilitate the expulsion of structurally diverse antimicrobials, thereby reducing intracellular drug concentrations to subtherapeutic levels [28,29]. The *tolC* gene, in particular, has emerged as a critical component of this network, aligning with its established role in mediating resistance to β-lactams, tetracyclines, and disinfectants in Gram-negative pathogens. Notably, the genomic co-localization of *tolC* with other efflux components on BA1′s plasmids indicates a modular genetic architecture that could be readily transferred to other enteric bacteria via horizontal gene transfer [30]. This mobility is further enhanced by the strain’s possession of insertion sequences and integrons, which are known to facilitate the rearrangement and amplification of resistance determinants [31]. Such genomic plasticity not only complicates containment efforts but also raises the specter of BA1-like strains emerging in human clinical settings, where similar efflux systems have been linked to treatment failures in urinary tract and bloodstream infections.

The virulence repertoire of BA1 is equally concerning, encompassing adhesins (e.g., *fimH*), toxins (e.g., *stx1*), and secretory systems (Types I–VI) that collectively augment its pathogenic potential [32]. Notably, the *fliC*-encoded flagellin, a critical factor in biofilm formation and host inflammation [33], was significantly upregulated in comparison to reference strains, as confirmed by both qRT-PCR and phenotypic assays. Under scanning electron microscopy, BA1′s biofilms demonstrated a dense, three-dimensional structure, with extracellular polymeric substances forming a physical barrier that likely hinders antibiotic penetration and immune clearance [34,35]. Our study reveals a key non-genetic resistance mechanism through biofilm formation, where dormant bacterial sub-populations evade antibiotics by reducing drug target activity, allowing survival during treatment. [36]. Once antibiotics are withdrawn, persister cells can revive and cause recurrent infections, contributing to the chronic persistence of BA1. This, combined with the strain’s efflux-mediated resistance, forms a dual-defense mechanism that makes conventional treatments ineffective, as seen in antibiotic-treated calves on the index farm showing no improvement. This reflects a broader *E. coli* evolution trend, driven by environmental stressors like antibiotic overuse, selecting for strains that enhance resistance, virulence, and persistence [37].

KEGG pathway analysis showed that BA1 is enriched in metabolic pathways, secondary metabolite biosynthesis, and microbial metabolism, highlighting its metabolic versatility for nutrient utilization and energy production. The enrichment in secondary metabolite synthesis suggests an increased ability to produce virulence factors like toxins and siderophores, aiding its pathogenicity and adaptability. These adaptations are crucial for its survival under antibiotic pressure and ecological success. While AMR surveillance often focuses on genetic resistance, our analysis of *E. coli* BA1′s secretory proteins and metabolic adaptations offers insights into its persistence and virulence. Notably, Type II and VI secretory systems in BA1 are linked to exporting toxins, biofilm components, and immune evasion factors, facilitating host colonization and environmental survival [38]. Metabolomic profiling showed increased siderophore production, carbohydrate metabolism, and stress response, highlighting the role of phenotypic plasticity in thriving under antibiotic pressure. This suggests that genomic resistance alone does not explain MDR strains’ success; secretion and metabolism are crucial. Future AMR studies should include functional proteomic and metabolomic analyses to understand adaptations, especially where antibiotics drive rapid evolution. Without this approach, interventions might miss physiological vulnerabilities like biofilm disruption or metabolic inhibition.

BA1′s evolution is closely linked to regional farming practices, where antibiotic use is common despite its role in AMR. Genomic studies place BA1 in the cgST23421 lineage, a group of farm-related *E. coli* known for plasmid-mediated resistance. This lineage’s link to calf diarrhea outbreaks indicates niche adaptation, likely due to routine antibiotic pressure [39]. The resistance genes of BA1 align with antibiotics like tetracyclines and sulfonamides used in the herd, suggesting direct selection as a key evolutionary force. This strain’s zoonotic potential is evident from its lethality in mice, causing severe sepsis and multi-organ damage. These findings support recent reports of MDR-*E. coli* spreading from livestock to farmworkers, highlighting farms as sources of resistance genes that can reach humans [40,41]. The study’s most alarming finding is the strong similarity between BA1′s MDR plasmids and those from human clinical isolates, indicating that resistance genes can directly transfer from animal to human pathogens. Antibiotic use in agriculture not only promotes resistant strains but also selects for mobile resistance elements like *IncF*-type plasmids, which can transfer resistance genes between different bacterial hosts, including those in the human gut. These plasmids can make treatable infections untreatable. While BA1 might be an endpoint, its mobile genetic elements pose a significant threat, perpetuating a “legacy of resistance” due to agricultural antibiotic misuse and worsening the global AMR crisis.

Addressing this threat demands a multifaceted strategy that integrates genomic surveillance, antibiotic stewardship, and innovative therapeutics [42]. Monitoring farm-related *E. coli* using WGS can detect resistance trends early, potentially preventing clinical crises. Stricter rules on non-therapeutic antibiotic use in livestock may reduce MDR strain development. Additionally, developing efflux pump inhibitors or biofilm disruptors could enhance antibiotic effectiveness, while phage therapy shows promise against MDR-*E. coli*. However, these solutions require global collaboration, as AMR is a cross-border issue affecting multiple species and ecosystems [43]. The persistence and evolution of MDR-*E. coli* in agriculture highlight a significant One Health issue. Our research shows that excessive antibiotic use in dairy farms not only selects for resistance but also leads to the emergence of highly adapted, hard-to-treat pathogens. This calls for a shift in veterinary practices from relying on antibiotics to preventive measures like vaccines, phage therapy, and microbiome modulation. Additionally, genomic surveillance of farms should be part of national AMR monitoring to detect and contain high-risk strains early. Future research should focus on understanding transmission dynamics between animals, humans, and the environment to support targeted interventions.

## 5. Conclusions

The BA1 strain exemplifies the intersection of multidrug resistance and hypervirulence within agricultural *E. coli* populations, primarily driven by the unregulated use of antibiotics. Its genomic composition, which includes efflux pumps, biofilm formation mechanisms, and mobile resistance genes, presents a significant threat to both animal and human health. Our findings highlight the urgent need for integrated One Health strategies to prevent the emergence and dissemination of such strains. Future research should focus on developing translational solutions, ranging from rapid diagnostic tools to non-antibiotic therapies, in order to prevent the onset of a post-antibiotic era in both veterinary and clinical contexts.

## Data Availability

The data that support the findings of this study are available from the corresponding author upon reasonable request. Results of sequencing raw data analysis and annotation results. Data quality control and pre-test results (MIC test). The genome sequence of the strain was submitted to the genome database of NCBI, and the GenBank accession number was obtained (chromosome: CP158510, plasmid A: CP158511, plasmid B: CP158512, plasmid C: CP158513, plasmid D: CP158514, plasmid E: CP158515).

## Supplementary Materials

The following supporting information can be downloaded at: https://www.mdpi.com/article/10.3390/biology14091257/s1, Figure S1: Genetic evolutionary analysis and resistance test results; Figure S2: Analysis of the risk of zoonotic transmission by mobile genetic elements; Figure S3: Genome Sequencing Quality Assessment; Table S1: Antibiotic susceptibility test results.
