# Supplementary material for: Whole-Genome Analysis of a Novel Multidrug-Resistant Escherichia coli Strain from Dairy Calves in Northeast China: Mechanisms of Antibiotic Resistance and Biofilm Formation
Source: Biology (Basel). 2025 Sep 12;14(9):1257. doi: 10.3390/biology14091257 (PMC12467046; doi:10.3390/biology14091257)
Supplement: Supplementary file 1 [file biology-14-01257-s001.zip › Separation and identification of results.pdf]

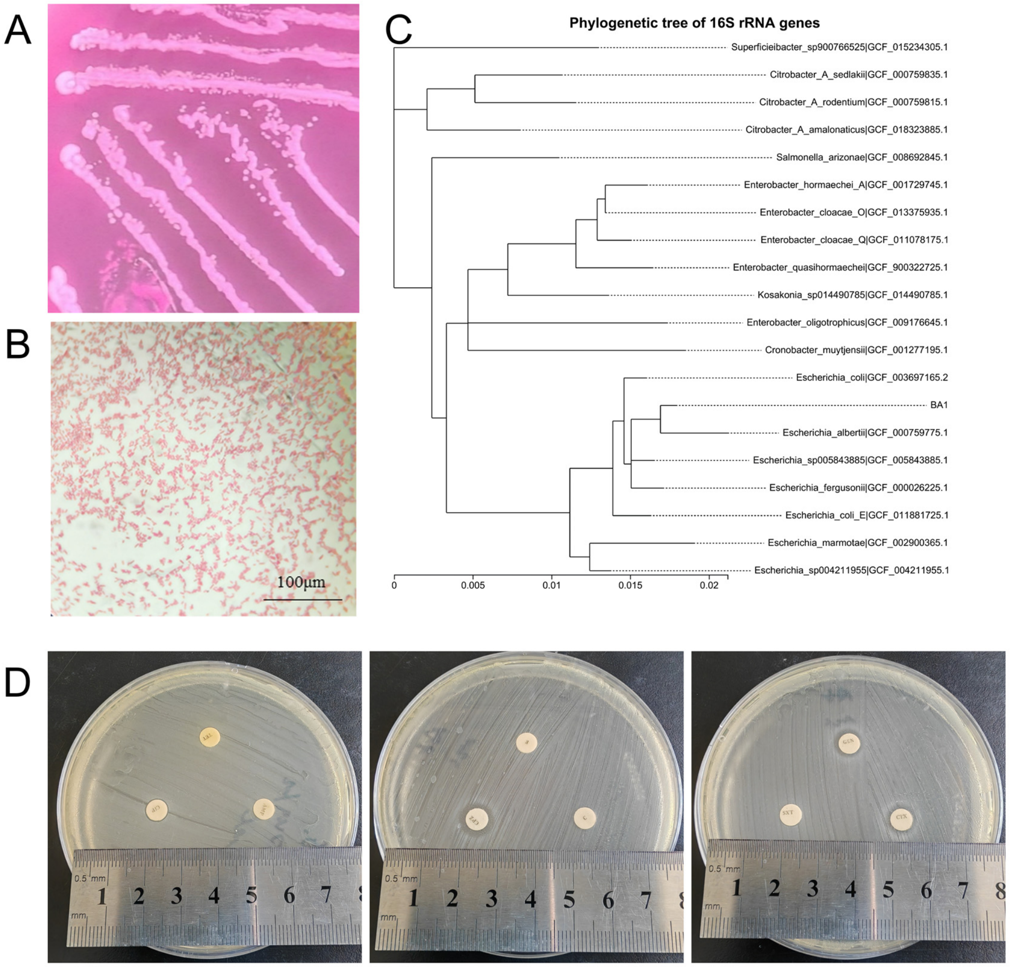

Figure S1. Genetic evolutionary analysis and resistance test results. A: Morphology of colonies grown in McConkey's medium after purification, B: BA1 microscopic image (Gram stain, 1000×), C: The 20 strains of bacteria that were closest to each other at the species level were selected based on 16S rRNA sequences by comparison with the NCBI database, and the phylogenetic tree was constructed by selecting the NJ (Neighbor-Joining) method through MEGA 6.0 software. D: Results of bacterial inhibition experiments by the paper slide method.

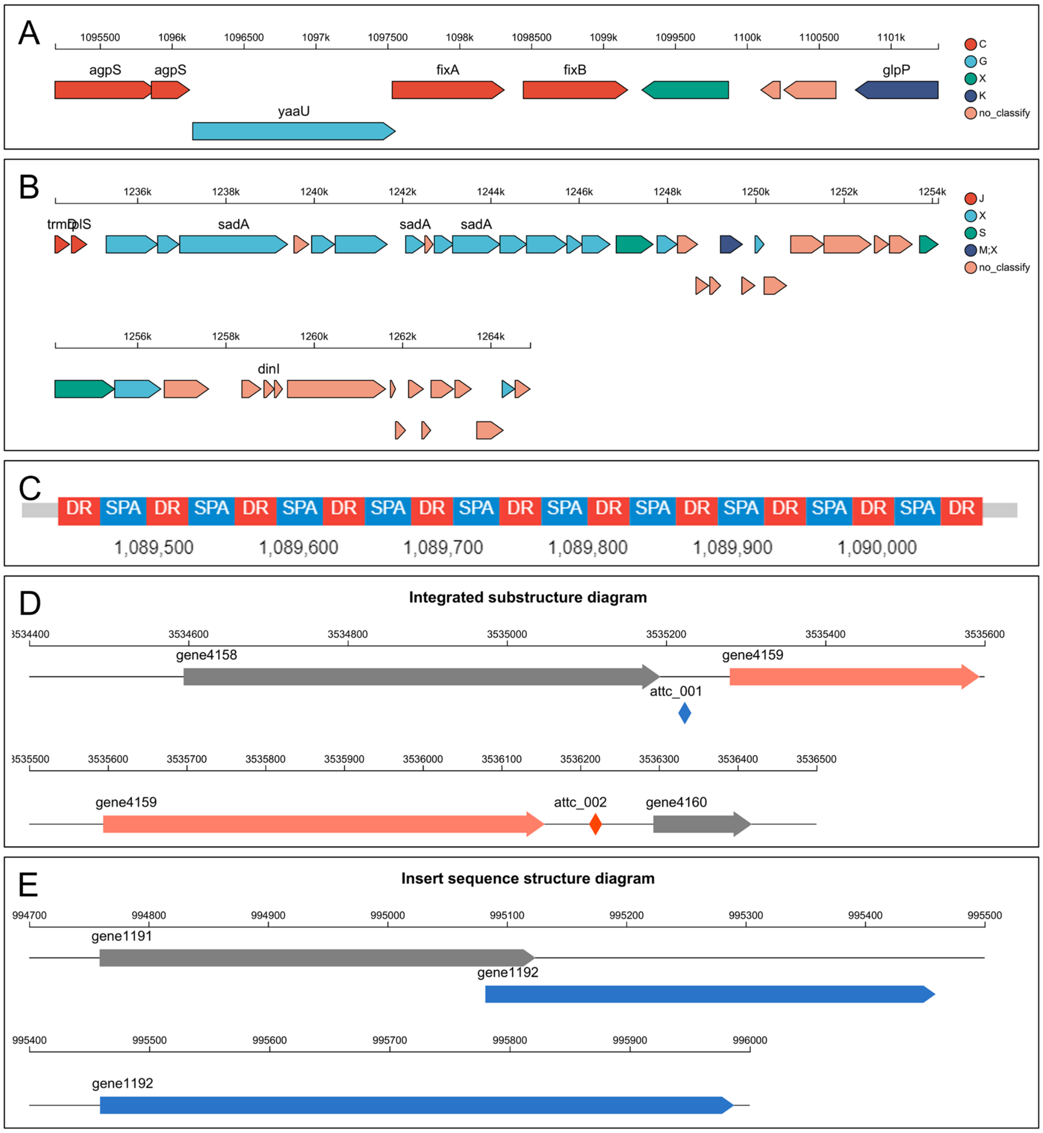

Figure S2. Analysis of the risk of zoonotic transmission by mobile genetic elements

A: Genome island analysis, B: Previous phage analysis, C: Statistical analysis of CRISPR-Cas. D: Integrator analysis, E: Insertion sequence analysis.

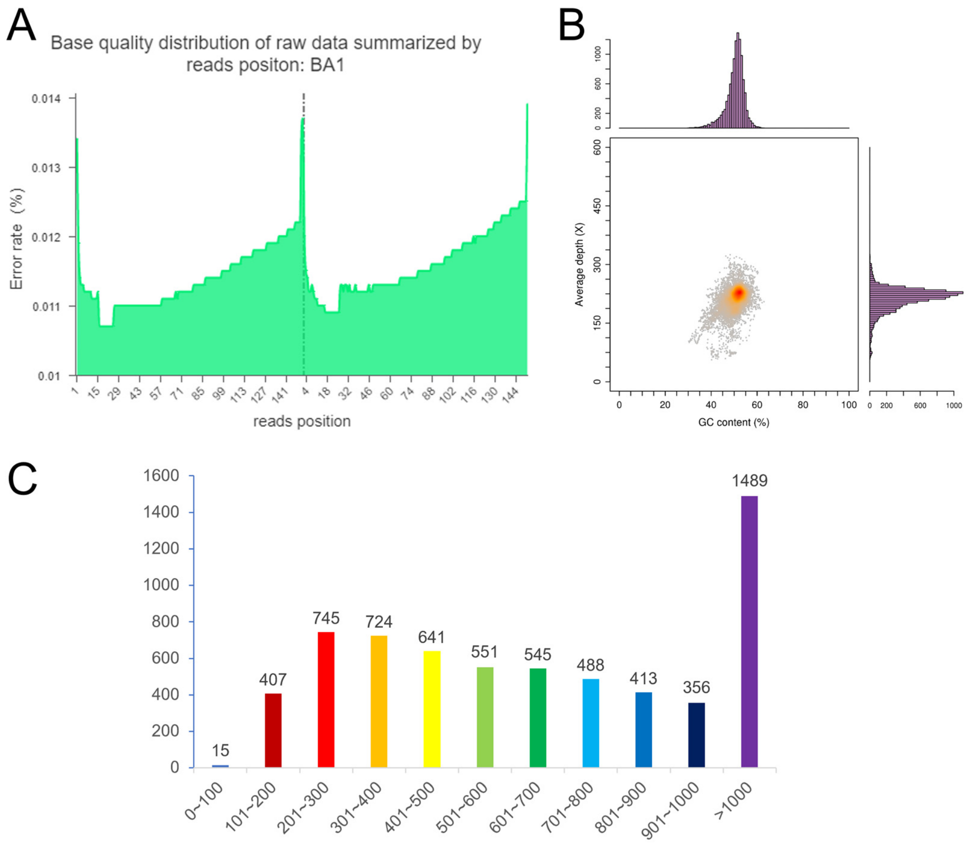

Figure S3. Genome Sequencing Quality Assessment. A: The horizontal coordinate is the base sitting position of the reads, indicating the sequential arrangement of bases on the reads from the 5' to 3' end; the vertical coordinate is the average error rate (%) of the bases of all the reads at the site. B: The horizontal coordinate is the GC content, and the vertical coordinate is the depth of coverage of the reads; the histograms on both sides, respectively, are the sliding-window frequency distributions of the GC content, and the sequencing depth. C: Number of large genes with different fragment sizes.

Table S1: Antibiotic susceptibility test results

Antibiotics	Inhibitory circle diameter (mm)	Result			
		R	I	S	
AMP	0	≤13	14-16	≥17	R
C	0	≤15	16-20	≥21	R
E	0	≤14	15-22	≥23	R
GEN	0	≤14	15-17	≥18	R
CPZ	11	≤14	15-18	≥19	R
TET	0	≤15	16-20	≥21	R
CIP	0	≤12	13-14	≥15	R
CTX	9	≤14	15-18	≥19	R
STX	0	≤15	16-20	≥21	R
